# Towards the Determination of *Mytilus edulis* Food Preferences Using the Dynamic Energy Budget (DEB) Theory

**DOI:** 10.1371/journal.pone.0109796

**Published:** 2014-10-23

**Authors:** Coralie Picoche, Romain Le Gendre, Jonathan Flye-Sainte-Marie, Sylvaine Françoise, Frank Maheux, Benjamin Simon, Aline Gangnery

**Affiliations:** 1 Laboratoire Environnement Ressources de Normandie, IFREMER, Port en Bessin, France; 2 Université de Bretagne Occidentale, Institut Universitaire Européen de la Mer, Laboratoire des sciences de l'Environnement Marin (LEMAR), UMR 6539 CNRS/UBO/IRD/IFREMER, Plouzané, France; The University of Wollongong, Australia

## Abstract

The blue mussel, *Mytilus edulis*, is a commercially important species, with production based on both fisheries and aquaculture. Dynamic Energy Budget (DEB) models have been extensively applied to study its energetics but such applications require a deep understanding of its nutrition, from filtration to assimilation. Being filter feeders, mussels show multiple responses to temporal fluctuations in their food and environment, raising questions that can be investigated by modeling. To provide a better insight into mussel–environment interactions, an experiment was conducted in one of the main French growing zones (Utah Beach, Normandy). Mussel growth was monitored monthly for 18 months, with a large number of environmental descriptors measured in parallel. Food proxies such as chlorophyll *a*, particulate organic carbon and phytoplankton were also sampled, in addition to non-nutritious particles. High-frequency physical data recording (*e.g.*, water temperature, immersion duration) completed the habitat description. Measures revealed an increase in dry flesh mass during the first year, followed by a high mass loss, which could not be completely explained by the DEB model using raw external signals. We propose two methods that reconstruct food from shell length and dry flesh mass variations. The former depends on the inversion of the growth equation while the latter is based on iterative simulations. Assemblages of food proxies are then related to reconstructed food input, with a special focus on plankton species. A characteristic contribution is attributed to these sources to estimate nutritional values for mussels. *M. edulis* shows no preference between most plankton life history traits. Selection is based on the size of the ingested particles, which is modified by the volume and social behavior of plankton species. This finding reveals the importance of diet diversity and both passive and active selections, and confirms the need to adjust DEB models to different populations and sites.

## Introduction

The blue mussel *Mytilus edulis* is common in Europe and North America and has been consumed by man for centuries. Aquaculture can be traced back to the 13^th^ century and now exceeds fishing due to its stability and the possibility it offers to regulate harvests. Production has been increasing for the last 50 years in response to the rise in mussel consumption and trade. This economic significance has drawn attention to *M. edulis*. Understanding the behavior and physiological responses of this species may help maximize its productivity through optimization of rearing strategies. Several models have been developed to describe mussel growth in relation to the environment. The Dynamic Energy Budget [1] theory has been the most successful of these models to date [2].

DEB models allow the quantitative description of energy acquisition and use in living systems. These models quantify energy fluxes through organisms, from energy uptake to its allocation to growth, maintenance and reproduction. As for other bivalve species [3], one of the difficulties in applying DEB models to *M. edulis* growth is to link trophic resources available in the environment with energy uptake (assimilation). In order to solve this problem, various modeling strategies have been used: the first and simplest of these consists in re-estimating the food-ingestion parameters for each studied location [4], [5]; this has allowed the DEB model to be adapted for low [6], [7] or high [8] seston conditions, for example. Different food proxies have also been tested. Chlorophyll *a* (chl a) has often been used, either as a raw input [9] or a refined input taking into account Chl a/C ratio [10]. Phytoplankton also gives good results [11], as does total particulate matter (TPM) [12]. Nevertheless, no consensus has been reached when comparing all these quantifiers [12]. In order to develop a more generic approach, other authors formalized more detailed processes for food ingestion that could incorporate several food proxies [13].

Knowledge is also needed about mussel feeding processes. Physiological aspects of food uptake have been thoroughly studied [14], [15], [16], [17], but data on the differential effects of food quality on growth are scarce. Recent studies tend to indicate that mass growth is significantly affected by diet quantity and quality [18], and that mussels may even modify their feeding behavior according to local food composition [19]. Until now, investigation focused on food physical properties, such as size, that could affect feeding behavior [20]. New techniques are being developed that give more detailed insight into the diet of *M. edulis* [21], [22], [23], [24] in terms of *e.g.*, types of food, phytoplankton species and diversity. Results are presently contradictory, however, even for the most generic points such as the comparative roles of diatoms and dinoflagellates [21], or the effect of particulate matter and other food sources (*e.g.*, detritus) [25]. This is partly due to local variability and lack of knowledge about feeding mechanisms. In the present work, we suggest that the DEB model can provide this type of information, as well as benefit from such data.

Inversing the model makes food reconstruction possible from growth observations. This has been done for several bivalves using different approaches: shell length can be mathematically related to ambient food using DEB assumptions [26]; successive approximates can be made for food [27] and the fit of different simulations obtained with different food proxies can be used as an indicator of their accuracy [12]. All these methods have their advantages and drawbacks and, until now, have focused on global food quantifiers. Here, we compare these three approaches at the scale of the phytoplankton species. This article is thus structured in three main steps: (1) we test two existing parameter sets for mussels, each representing a different way of studying food; (2) we then develop and apply our own food reconstruction methods and (3) use results to deduce general properties for food quality assessment.

## Materials and Methods

### Study area

**Figure 1:**
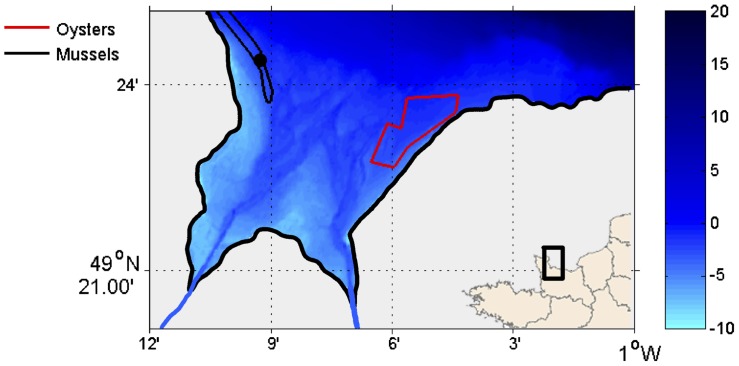
Location of the study area in the Bay of Veys.

Mussels were raised on the French coast of the English Channel in the western part of the Bay of Seine, northwest of the Bay of Veys and south of Utah Beach (49°24.369′N/1°09.230′W) (Fig. 1). The Bay of Veys covers 37 km^2^ and has a semi-diurnal macrotidal regime with maximum amplitudes ranging from 2.5 to 7 m during neap and spring tides respectively. Freshwater inputs enter this area from the Carentan and Isigny channels, supplied by four rivers, dominated by the Vire, which contributes up to 40% of total flow [28]. Jouenne *et al.* [29], [30] described the primary production dynamics at different timescales in this bay. This small catchment area is characterized by relatively high chl a content and low turbidity compared with other estuaries, making it an intermediately productive ecosystem. Nutrient deficiency is debated: while some authors consider that their input is sufficient in the Bay of Veys [30], others hypothesize that nitrogen might be limiting during spring [10]. Anthropogenic activities seem more important than climatic conditions in explaining temporal variability in ecosystem functioning. There is a significant level of shellfish farming in this area: cultured *Crassostrea gigas* amounted to 2262 tons on Utah Beach in 2006, accounting for 14% of oyster production in whole of the Bay of Veys; *M. edulis*, at about 1332 t, represented about 85% of total mussel production in this area.

### Field measurements

Mussel seed used for the experiment was collected during spring 2009 in La Plaine-sur-Mer (French Atlantic coast), and first transferred to pregrowing structures in Agon (west coast of Normandy). Pregrown mussels (20.99±1.85 mm and 0.79±0.04 g) were finally installed on Utah Beach in mid-September 2009. This site is located at around 1 km offshore and could be accessed from land at low tide for mussel sampling and by sea for hydrological measurements at high tide. Tide variation in this area led to daily emersion of the mussels lasting around 39±5% of the day. All structures were located on private sites lent by professional oyster or mussel farmers. These sites are usually used for shellfish farming and the experiment was therefore not considered to alter the environment, flora or fauna. Mussels were put in 18 plastic baskets (35 cm in height and a triangular section of side 20 cm). The baskets were installed on the middle height of 3 adjacent poles at a rate of 6 baskets per pole. Poles were separated by about 1 meter. They are the common rearing structure used for mussel culture in Normandy. Sixty mussels were placed in each half-basket, giving an initial available volume of 50 cm^3^ per individual. Ninety-six individuals were separated in 9 specific nets for individual shell length monitoring. These nets were distributed in 3 baskets at a rate of 3 nets per basket. Baskets were installed on a fourth adjacent pole. Monthly sampling began in January 2010 and continued for 18 months. Each sampling date comprised two sets of measurements: one from mussels that were alive throughout the study and one from mussels that were sacrificed. In the first set, the shell length (L in mm) of the 96 identified mussels was determined *in situ* using a manual caliper (FACOM, accuracy: 0.02 mm), allowing the acquisition of individual trajectories. For the second set, the content of one randomly selected basket was sampled at each date and brought back to the laboratory. Forty of the live mussels were randomly selected, measured using a digital caliper (MITUTOYO, accuracy: 0.02 mm) and sacrificed in order to separate flesh and shell. Dry flesh mass (DFM) was measured after a complete freeze-drying cycle (METTLER TOLEDO Balance, accuracy: 1 mg). Based on the assumption that average flesh mass is proportional to cubed mussel length (eq. 1), masses were corrected for size differences on the basis of the length obtained from the individual monitoring [31].
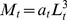
(1)where M_t_ is the reconstructed flesh mass, L_t_ is the measured length from the non-destructive sampling at date t and a_t_ is the coefficient relating length and mass, calculated with the destructive sampling. An example of such relation between mass and length is given in Fig. S1. This is different from the DEB formulation relating length and mass with a constant parameter set (eq. 13 in Text S1). Here, a_t_ is recalculated at each sampling date.

The two sets of mussels, corresponding to two different processes, were necessary because of the invertebrate nature of the mussels. Neither body length, nor flesh mass can be accessed without killing the animal. However, individuals should not be killed as their continuous monitoring alone can accurately assess growth. Indeed, the use of different individuals at each sampling date may introduce a bias: two different basket samples can show growth variations that should not be taken into account in the model. As a consequence, we used a first set of sacrificed mussels to calculate a proxy of the relation between shell length and flesh mass. We then used the shell length, the only measurement that we could obtain from the second set of non-sacrificed mussels, to reconstruct the dry flesh mass of the individuals. As a consequence, the dry flesh masses that are used as inputs in the model are a proxy of the masses of the same individuals at each sampling date.

Hydrological data (water temperature, water depth and salinity) were continuously recorded with an autonomous NKE data logger STPSO2-SI placed in a basket. Two data loggers were specifically dedicated to the experiment, used in rotation and changed every month. Observations were checked for outliers or drift, cleaned when necessary and filtered to account for immersed values only. As a consequence, temperature measurements were used only when they corresponded to immersion time. Metrological verifications were made before each deployment in the field. The frequency of data acquisition was set at 10 minutes.

Hydrobiological parameters were assessed fortnightly for a total of 33 sampling dates over 18 months. All water samplings were performed from the boat within the two hours around high tide. For nutrients (ammonia NH_4_^+^, silicates Si(OH)_4_, phosphates PO_4_^3−^ and nitrates-nitrites NO_2_+NO_3_^−^), samples were taken 1 m below the surface using a Niskin bottle. Pre-filtrations were made on board with a 48 µm mesh for all nutrients plus a 0.45 µm mesh for silicates. Back in the laboratory, analyses were done with a Technicon Autoanalyzer III, according to the method described in [32]. For the other parameters, water samplings were performed by hand at around 50 cm below the surface. Particulate matter samples were filtered in duplicate through pre-combusted (450°C for 1 h) and pre-weighed Whatman GF/F filters, rinsed with distilled water to remove salts and stored at −20°C until analysis. Filters were dried at 70°C for at least 2 h and weighed for total particulate matter (TPM, mg dry mass l^−1^). Inorganic matter (PIM, mg ash dry mass l^−1^) was given by the mass of ash remaining after burning at 450°C for 5 h. Organic matter (POM, mg ash free dry mass l^−1^) was given by losses at ignition. PIM and POM values are available from February to October 2010. In order to estimate trophic resources potentially available for mussels, phytoplankton biomass (chl a and pheopigment concentrations, µg l^−1^) and composition (cell abundances of micro-, nano- and picoplankton, cell number l^−1^) and particulate organic carbon (POC) and nitrogen (PON) were determined. For chl a and pheopigments, samples were filtered in duplicate through Whatman GF/F filters, which were frozen at −80°C for up to a month. Pigments were extracted in 90% acetone for 12 h and analyzed with the Lorenzen spectrophotometric method described by [33]. For POC and PON determination, water samples were filtered in duplicate through Whatman GF/F filters, rinsed with sodium sulfate and analyzed with a CHN analyzer according to [33]. Additional filtrations were also made for blank materials [33]. Water samples were fixed in a Lugol's solution for phytoplankton determination. The portion of phytoplankton between 20 and 200 µm in size was identified and counted in 10 ml tanks with the Ütermohl method [34] using a phase-contrast inverted microscope (Olympus IMT2 or IX71). The same analyst conducted the identification process for the entire experiment in order to maintain a consistent account of species evolution. Accuracy started at the family level and could reach species or groups of species depending on the plankton morphology. In agreement with the analyst, the level of detail was coarser in the post-processing than during the experiment: 53 out of the 77 initially identified groups of plankton were finally used. This approach discarded potential mistakes in the identification process. For pico- and nanoeukaryotes, as well as the bacteria *Synechococcus* and *Cryptophyceae*, water samples of 1 ml were fixed in 1.8 ml cryotubes with electron microscopy grade glutaraldehyde at a final concentration of 1% (vol/vol) and immediately stored in liquid nitrogen for a few months [35]. Samples were then counted by flow cytometry according to [36].

Finally, hourly rainfall measurements, wind direction and velocity and irradiance were provided by Météo France for the Sainte-Marie-Du-Mont station, located 6 km southwest of our sampling site.

### Dynamic Energy Budget model

The model used in this study is based on the Dynamic Energy Budget (DEB) theory [1]. This model quantifies growth and energy allocated to reserves, structure and reproduction as a function of two forcing variables: water temperature and food availability. This type of model has been widely applied to the study of bivalve energetics [3], [4], [6], [10] and has been used to predict *M. edulis* growth and reproduction [5], [7], [9], [12], [13]. The main equations are given in Text S1. Implementation was taken from Rosland *et al.* [6], including a possible decrease in somatic mass during starvation (eq. 12 in Text S1), and adapted to take into account additions and adjustments concerning food assimilation and energy processing from Saraiva *et al.* [13] (eq. 1 to 4 in Text S1). Briefly, the model describes the energetics of an individual through the dynamics of three state variables: reserves (E), structure (V) and energy allocated to reproduction (E_R_). Energy is taken from the environment and fuels the reserve compartment (eq. 1 to 6 in Text S1). A constant fraction κ of this energy is allocated to somatic maintenance and structural growth and the remaining 1-κ is allocated to maturity maintenance, development (in juveniles) and reproduction (in adults) (eq. 7 to 10 in Text S1). The energy content of the reproductive buffer is liberated at spawning. In the present application, spawning dates are forced and correspond to drops in dry flesh mass (DFM) [6]. Such a formulation is less flexible than an implementation based on gonado-somatic index (GSI) and water temperature thresholds [5] but was chosen for simplicity purposes. The reproductive buffer is assumed to be totally emptied at spawning, according to Sprung *et al.* [37].

The Arrhenius law was used to correct all rates for temperature. Main parameter values are given in Table 1. Recent food intake developments were included, requiring a calibration based on the carbon content of the particles [13]. In the absence of further knowledge, a conversion-to-carbon factor λ was added and calibrated for each food source (eq.1 in Text S1); some conversion factors are shown in Table 2. Additionally, due the same lack of knowledge, assimilation efficiency γ was assumed to be constant and equal to 0.75 [6].

**Table 1:** DEB parameters and values.

Symbol	Description	Value	Unit
TA	Arrhenius temperature	7022	K
[ 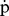 _M_]	Volume-specific maintenance costs	11.6	J d^−1^ cm^−3^
[E_m_]	Maximum storage density	1438	J cm^−3^
[E_G]_	Specific cost for structure	5993	J cm^−3^
κ	Fraction of reserves spent on somatic growth and maintenance	0.67	-
δ	Shape parameter	0.297	
{ 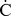 _Rm_}	Maximum surface area specific clearance rate	96	L d^−1^ cm^−2^
{ 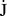 _a_F}	Algal max. s.a. specific filtration rate	0.00048	mol C d^−1^m^−2^
{ 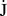 _i_F}	Inorganic material max. s.a. specific filtration rate	3.5	g d^−1^ cm^−2^
ρ_a_	Algal binding probability	0.99	-
ρ_i_	Inorganic material binding probability	0.4	-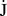 _a_I	Algal max. ingestion rate	13000	mol C d^−1^
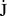 _i_I	Inorganic material maximum ingestion rate	0.11	g d^−1^
C	Conversion factor	697000	mol J^−1^
AE	Assimilation efficiency	0.75	-

Values were taken from Saraiva (2011a) and adapted to allow different food proxies.

**Table 2:** Food proxy calibration and use in the model.

Food source	Conversion parameter	Deviation (%)
Chl a	7.9×10^−6^ mol C.(µg Chl a)^−1^	23.2
Chl a × richness	4.6×10^−7^ mol C.(µg Chl a)^−1^	21.2
POC	8.3×10^−8^ mol C.(µg C)^−1^	37.7
Phytoplankton	3.6×10^−6^ mol C.(Cell)^−1^	35.8
Chaetoceros	1.8×10^−9^ mol C.(Cell)^−1^	18.6

Food proxies had to be converted to mol C to adapt them to the rest of the model. Deviation corresponds to the relative difference between simulated and observed DFM, as described in eq. 2.

### Simulation and validation

Simulations were run with Matlab (Matlab R2012b), using the implementation of Rosland et al. [6]. Input daily temperature was the daily average of water temperature, measured during immersion time. Daily available food was linearly interpolated from the fortnightly observations. Chl a, abundance of micro-, nanophytoplankton and even smaller species like bacteria or *Synechococcus* (alone or as a group), POC and TPM were tested as model inputs one after the other. Composite variables were also tested by balancing chl a or phytoplankton abundance with the corresponding richness (number of species) or evenness (calculated as the ratio of the Shannon diversity index and the natural logarithm of the number of species). To account for inedible material, PIM was approximated as 80% of TPM. This is the maximum ratio of PIM to TPM that was measured during the experiment and was used to reduce assimilation [13]. Initial mass was the average mussel mass at the beginning of the experiment. Shell length was calculated accordingly.

The effect of different food sources on growth was assessed by deviation *d* (eq. 2). This calculates the distance to mass measures.
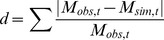
(2)where *d* is the deviation of the simulation, and *M_obs,t_* and *M_sim,t_* are the respective observed and simulated DFM at observation time t.

A non-linear optimisation method (Nelder-Mead) was applied in the auto-calibration, which searched iteratively for the λ values that minimised the deviation *d*.

### Food reconstruction

An inverse method was used to assess the quality of our food sources. Shell length and DFM were used to compute the corresponding functional response over the 18-month experiment. This response is taken as a function of both food availability and digestibility.

The first step was to reconstruct the evolution of the functional response over time from individual shell length time series, using a reversed DEB model as described in [38]. Briefly, temperature and shell length are taken as inputs of this reversed model to calculate the corresponding functional response. Different individual measurements of length are averaged and interpolated using a spline function so that the reversed model applies on the same daily time step as the standard one. The reconstructed functional response is based on the same equations and is therefore theoretically exact.

An iterative method was then used to compute a functional response corresponding to mass variation. The functional response was used with the modeling of Rosland *et al.* [6] (eq. 5 and 6 in Text S1) including the non-food related parameters that have been described by Saraiva *et al.* [13]. An initial functional response was built to vary randomly between 0 and 1. This was then used as an input for the simulation, skipping food ingestion and assimilation to affect growth directly. To do this, the classic DEB formulation was used ([6]), adjusting the maximum assimilation rate with the functional response term. Simulated growth was then compared with observed growth. For each time step between first and last sampling dates, the functional response was respectively increased or decreased as long as the simulated mass was below or above observed mass within a 1% range, and functional response was above 0. These stringent conditions ensured that variations were respected over time while maintaining a physiological sense. A functional response above 1 may indicate a bad parameter value for the maximum assimilation rate, which was expected from the results of the first simulations. On the contrary, a negative functional response can only indicate “negative” assimilation, which does not make sense.

Both reconstructed functional responses were then scaled by their maximum to vary between 0 and 1, and smoothed with a 4-day moving average to remove modeling bias.

### Food quality assessment

The contribution of each food source was evaluated for each sampling date after being transformed with a Holling-type II function (eq. 3).
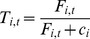
(3)where *T_i,t_* is the transformed food source *F_i_* at time *t* and *c_i_* is a scaling coefficient, linked to saturation or satiation. This parameter regulates food absorption.

This transformation was meant to avoid signal distortion and to homogenize units between different food sources (phytoplankton abundance may vary between 0 and 10^6^). It remains close to the usual DEB model formulation and helps to scale different food values. The same scaling constant was used for both functional responses (length and DFM) as mussels ingest food in the same way, with possibly different allocation. Values of both signals were similarly extracted. A linear combination of processed food measures was then used as a proxy for functional response signals. Each sampling date is thus characterized by a system of two equations (eq. 4).
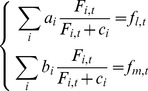
(4)Where *a_i_* and *b_i_* represent the contribution of the food source *F_i_* to growth in length or mass, respectively, and *c_i_* is the scaling coefficient, which should be understood in this context as an index of the quantity of available food above which the contribution to total food input is maximal. It can be seen as the intensity with which mussels react to food presence: a lower *c_i_* helps reaching *a_i_* and *b_i_* with lower food concentrations. *f_l_* and *f_m_* are the functional responses corresponding to length and mass, respectively.

Thirty-three sampling dates were available, which gave 66 equations. Each food source needed to be described by 3 parameters (*a_i_*_,_ *b_i_* and *c_i_*). An identifiability analysis ([39], used in [40], [41], [42]) showed that all three of these parameters could not be determined at the same time with a sufficient number of plankton species to study the ecological characteristics (see Text S2, Fig. S2 and S3 for more details about model assessment). *c_i_* parameters were thus fixed, corresponding, for each species, to the median value of the abundance when the species was present in the field. This is close to the value that is obtained for *X_k_* with the DEB model when using a single plankton species as the food input. The number of species for which *a_i_* and *b_i_* can be calculated is a trade-off between the condition index of the matrix model (indicating the quality of the formulation) and the final model error: in our study, 30 plankton species or groups of species was the maximum we could take into account while maintaining a reasonable condition index (3.3×10^3^). These were chosen as the most abundant species that were also large enough to be efficiently retained [20].

Resolution was performed with Matlab's active-set sequential quadratic programming under constraints (Matlab R2012b). Plankton species used in hatchery were assumed to have a positive impact on growth (*a_i_* and *b_i_* are positive for *Chaetoceros, Skeletonema costatum, and Naviculaceae* for instance [43]). Initial *a_i_* and *b_i_* followed a uniform random distribution between −1 and 1, complying with the above constraints.

*a_i_* and *b_i_* were then transformed according to eq. 5 to represent relative contributions to total diet value.
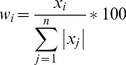
(5)where *w_i_* is the relative contribution of the food source *i* corresponding to the coefficient *x_i_* (*a_i_* or *b_i_*) and n is the total number of food sources.

### Statistical analysis

Relative contributions of each plankton species to total diet value were linked with plankton characteristics. Several databases ([44], [45], [46] and M. Schapira's personal atlas) were collated and completed with data from the literature (see Table S1). Family was the first classification criterion, separating diatoms, dinoflagellates and others. Among diatoms, pennate and centric species were differentiated. Biovolume, surface area and their ratio were taken from [46], using the median value of the observations in our study area. The difference between smaller and larger species was qualitatively assessed by the plankton analyst to account for local variability. Biovolume and area were then log-10 transformed. Cell shape was also extracted from [46], using the conventions in [47]. Habitat values classified plankton according to their preference for coastal or pelagic areas ([28], [45]). Habitat was turned into an ordinal variable ranging from 1 to 3, 1 characterizing species that are specifically found in a coastal brackish environment, including those coming from freshwater inputs and 3 characterizing species that could mostly be found in the ocean. This was decided using classifications by [48], [49], [50] and [51] and evaluation of salinity and eutrophication tolerance. Plankton social behavior qualifies colony frequency. For some species, this has been quantified as the mean number of cells per colony. When this number is below 1, the species is considered as single. Above, it is considered colony forming.

Explanatory variables were chosen based on a stepwise approach using the AIC relative change as the selection criterion (LinearModel.stepwise routine in Matlab 2012b). For contribution based on DFM, social behavior and biovolume were the only relevant parameters while contributions based on length were influenced by social behavior only. To be consistent, an ANCOVA (analysis of covariance) was performed for both relative contributions, taking into account both explanatory variables.

## Results

### Mussel growth

Out of the 96 individually-monitored mussels, 22 individuals died during the experiment and shell-length measures of 18 individuals decreased at least once during the experiment. These trajectories were removed before data processing. Monitoring data obtained with and without sacrifice were consistent.

**Figure 2:**
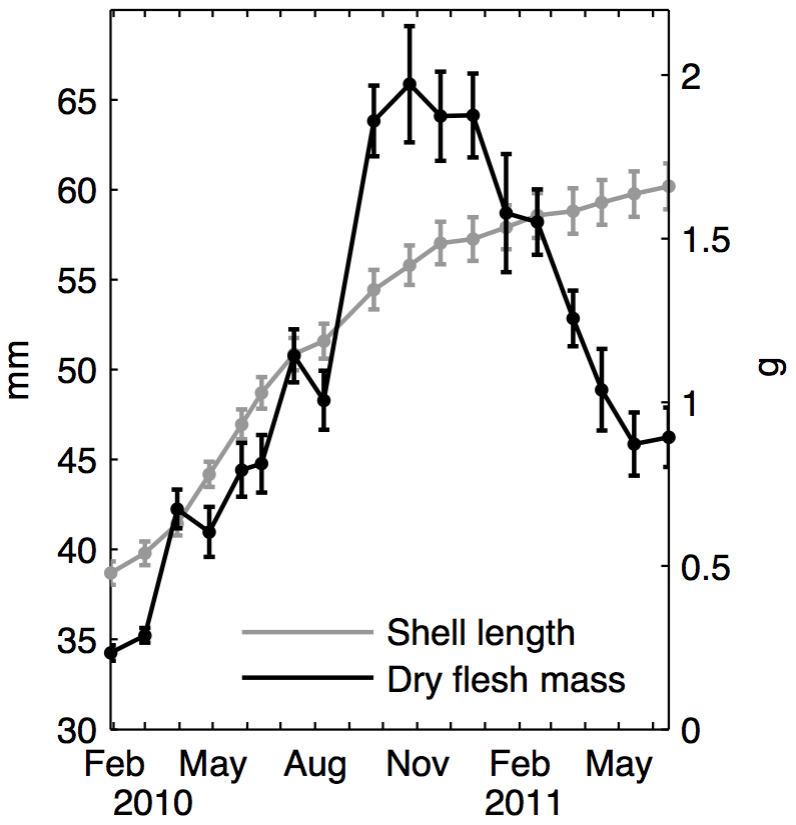
Observed mussel growth during the experiment. Shell length (grey) and dry flesh mass (DFM) (black) mean values and deviations correspond to monitoring with sacrifices, corrected for sampling bias.

Mussel growth is shown on Fig. 2. During the experiment, shell length increased from 36 to 60 mm. Maximum relative length increase took place in April 2010 with a 7% gain. Shell length increased by 1.7 mm per month during 2010, while 2011 was characterized by a length gain of around 0.45 mm per month. Mass observations can be divided into two main periods: 2010 was characterized by a mass gain followed by a period of loss during year 2011. DFM increased by 1.7 g between February and October 2010. This high growth mainly took place during March, with a growth rate of +13 mg d^−1^ and between August and September (+19 mg d^−1^). Mass was then stable until the end of the year. DFM was halved between January and May 2011.

Mussel length and mass growth were uncoupled: periods of maximum growth did not occur at the same time for shell length and flesh. In addition, the drop in flesh mass was obviously not reflected by shell length.

### Environmental conditions

**Figure 3:**
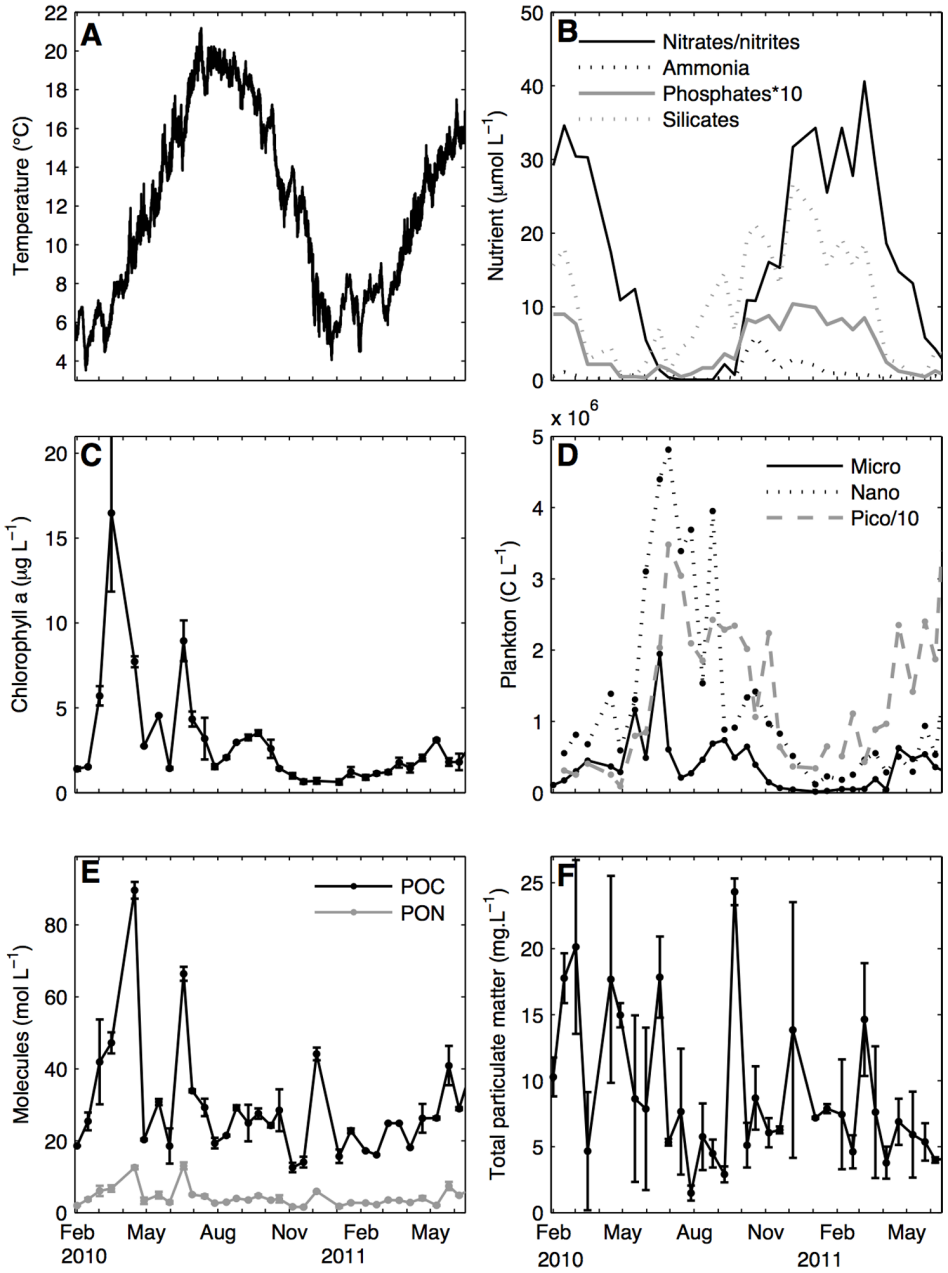
Observed environmental descriptors during the experiment. Average daily sea water temperature (A), nutrients (B), chlorophyll a (C), abundance of different sizes of plankton (D), particular organic carbon and nitrogen (E) and total particulate matter (F) were measured in 2010 and 2011.

Variations of the main environmental descriptors over the studied period are shown in Fig. 3. During 2010, water temperature (Fig. 3A) varied between 3°C in February and 21°C at the beginning of July. During 2011, it varied between 4.4°C at the end of January and 17.6°C at the beginning of June. Average temperature from February to mid-June was 9.4°C in 2010 vs. 10.8°C in 2011. Water temperature varied 1.1±0.6°C within a day, with a minimum variation of 0.2°C and a maximum of 3.3°C, which is low enough to consider temperature to be constant over a day in the DEB model. Rainfall, irradiance and nutrient concentration dynamics (Fig. 3B) were not significantly different between the two years (Kolmogorov-Smirnov test, p>0.05) and patterns were consistent with previous records on this point.

Chl a dynamics can be divided into two periods (Fig. 3C). In 2010, two peaks appeared, in mid-March (16.5 µg L^−1^) and mid-June (8.9 µg L^−1^), followed by a longer period with a chl a level stabilized around 3 µg L^−1^ for a month at the end of summer. It reached its minimum value of 0.6 µg L^−1^ in December. Conversely, the first six months of 2011 were marked by a low chl a concentration for this period of the year. It only reached a maximum of 3.1 µg L^−1^ at the beginning of May and therefore showed a low spring plankton biomass. Between February and June, chl a was on average 66% higher in 2010 than in 2011. The maximum difference was in the intensity of the spring bloom.

Nanoeukaryote and phytoplankton dynamics were similar to one another (Fig. 3D). Nano- and microplankton abundance were low during the first four months, then tripled in mid-June 2010 to reach almost 5×10^6^ C L^−1^ and nearly 2×10^6^ C L^−1^ respectively. End of summer and autumn were also characterized by two smaller peaks of abundance. Winter had a low concentration of microorganisms. In 2011, phytoplankton growth resumed in April, resulting in a 13-fold increase in biomass. Nanoeukaryote abundance began increasing significantly from mid-May. Nanoeukaryotes were more abundant in 2010 than in 2011 throughout the common experiment period. Picoplankton abundance was about one order of magnitude higher than nanoeukaryote abundance but seemed to follow the same patterns. It reached a peak in July 2010 and remained high until November. After a sharp decrease, it bloomed again from April 2011, with values comparable to those observed in 2010. Overall, it may represent 86% of total chl a production. No difference was significant between the two years for the picoplankton populations.

Richness varied between 11 and 31 species in 2010, with a median value around 20, while it varied between 7 and 20 in 2011 with a median value of 14 and a standard deviation around 2.9 for both periods. It was positively correlated with abundance (R^2^ = 0.31, p = 0.08). Evenness varied between 0.27 and 0.86 with a deviation of 0.17 in 2010, and varied between 0.33 and 0.74 with a deviation of 0.13 in 2011; it was negatively correlated with total abundance (R^2^ = −0.64, p<0.05). Chl a concentration could be related to these dynamics. The first chl a peak cannot be totally explained by phytoplankton or nanoeukaryote abundance but is confirmed by pheopigment concentration on the next sampling date. Phytoplankton counts explain the dynamics of last two blooms. In July, *Asterionellopsis glacialis* accounted for 76% of the total phytoplankton bloom while *Chaetoceros* amounted to 77% of the September phytoplankton biomass.

Chemical compounds did not vary as much between the two years as the variables mentioned above (Fig. 3E). POC ranged between 12.6 and 89.6 mol C L^−1^ during 2010, and between 16.1 and 40.9 mol C L^−1^ during 2011. PON ranged between 1.6 and 13.0 mol N L^−1^ during 2010, and between 1.7 and 7.4 mol N L^−1^ during 2011. Considering only the February–June period to compare the two years, there was a 33% decrease in POC and PON in 2011. This is only half the difference in chl a between 2010 and 2011. POC and PON are not only due to algal presence but also to detritus and river inputs.

Water quality was also impacted by TPM (Fig. 3F) as part of it is inorganic and may decrease food quality ([13]). TPM was highly variable (CV = 63%), ranging from 1.49 mg L^−1^ in July 2010 to 24.3 mg L^−1^ two months later. 2011 showed less extremes with only one peak around 14.6 mg L^−1^ in March. The lower concentrations observed in 2011 should nevertheless be interpreted with caution as the strong northeasterly wind that predominated during this period may have suspended many more particles than the levels recorded at low frequency.

Relating food abundance and growth patterns is a first step towards simulations. Usual food proxies such as chl a and plankton abundance decreased in 2011, which can partly explain the observed mass loss. However, mass gain timing in 2010 cannot be totally explained by these food sources. Indeed, the first mass increase in March 2010 cannot be related to plankton abundance, but can be related to a chl a peak. Conversely, the extent of the mass gain in September 2010 cannot be related to a comparable chl a increase but it can be linked to a planktonic bloom. In contrast, the plankton bloom in June 2010 was not related to any great increase in mass. From the other point of view, POC and PON do not decrease enough in 2011 to totally explain the mass loss.

### DEB model

**Figure 4:**
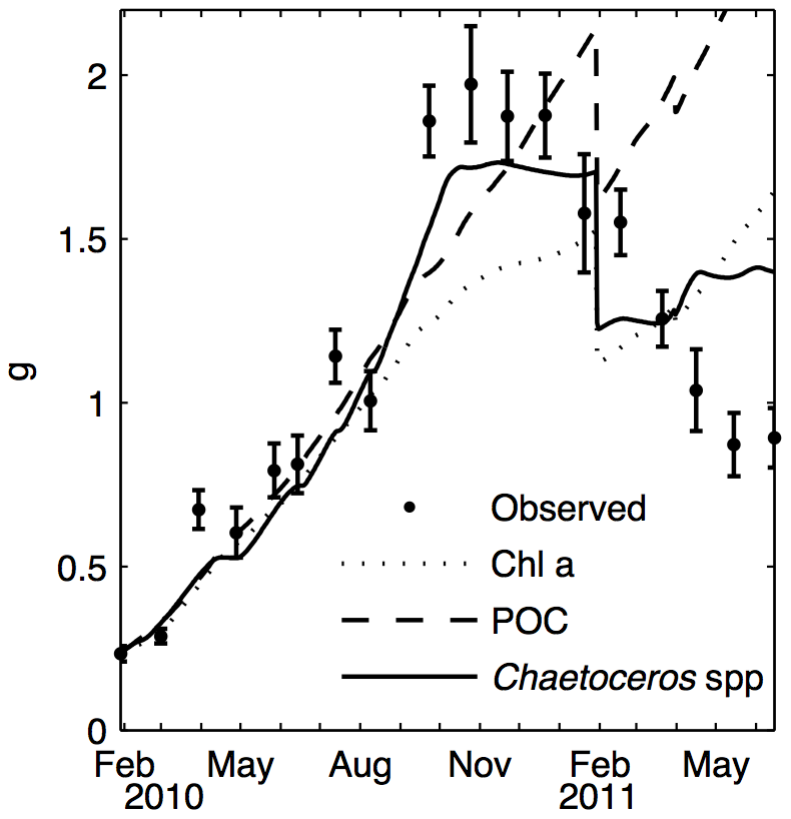
Comparisons of observed and modeled dry flesh mass (DFM) with different food inputs. Observations (dots) and DEB simulations (lines) are based on *in situ* measurements of food sources, with chl a (dashed line), POC (dotted line) and *Chaetoceros* spp. separately (solid line). The latter produced the best fit.

The first simulations with the DEB model did not provide satisfactory results, especially when chl a and POC were used as food proxies, as shown on Fig. 4 and Table 2. The model failed to represent both mass gain and loss. The two steep slopes of the growth curve in March and August 2010 could not be reproduced and led to underestimates of DFM at the end of the growth season and during winter. On the contrary, food was always sufficient in 2011 to allow growth or, at least, to avoid the mass loss observed in the field. A different set of parameters (from [6] and [13]) may lead to different decrease due to spawning. The most satisfactory results were obtained using the *Chaetoceros* genus alone, which cannot represent the reality of mussel nutrition. However, when chl a was balanced by species richness, the second best fit was obtained with a total deviation of 21%; these contrasting results led us to consider the plankton species in more detail.

**Figure 5:**
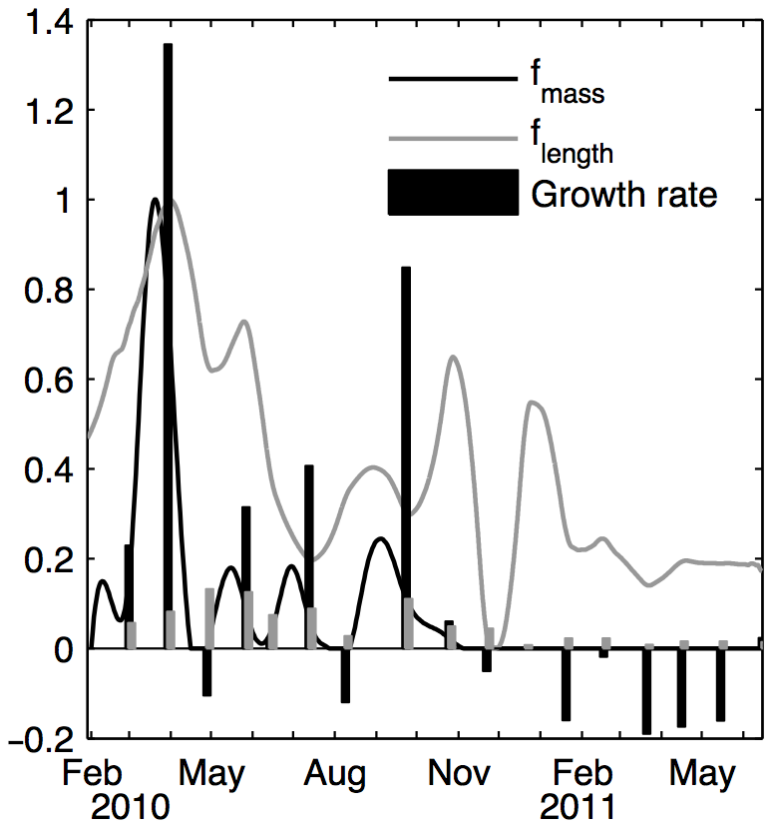
Comparisons of functional responses obtained with shell length (grey) and DFM (black) variation (see text for functional response computation). Bars represent the daily relative growth rates between two points. Both functional responses were standardized to vary between 0 and 1.

Figure 5 shows the functional responses computed with the reverse DEB model and compared to length and mass growth rates. Length and DFM do not show the same patterns, as their increase and decrease do not match.

**Figure 6:**
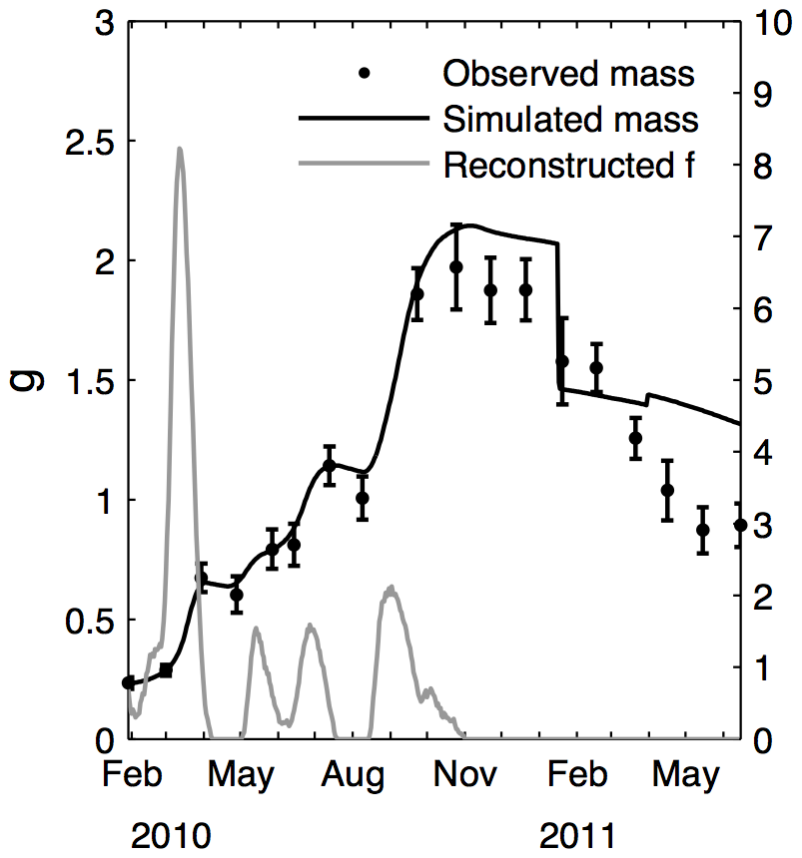
Comparisons of observed and modeled DFM with the reconstructed functional response. Functional response without standardization (grey) was obtained with DFM observations. Simulated DFM is shown by solid lines, observations are shown by dots.

Figure 6 shows the difference between observed and simulated DFM, using the corresponding reconstructed functional response. This highlights problems in the parameter sets that could not be totally remediated by adjusting food input. Food availability is not the only explanation of these variations, as a functional response of more than one is necessary to reach the masses observed in 2010. This is especially true in March where the simulated functional response can reach 8. Mass loss was underestimated when no food was input in 2011.

### Food quality

Table 3 shows the contributions of the 30 different plankton species to the reconstructed function responses based on length and soft tissue growth. Of the tested plankton groups 77% were diatoms.

**Table 3:** Phytoplankton groups and contributions for flesh- and length-based growth.

Plankton group	Contribution to flesh growth (%)	Contribution to shell length growth (%)
*Asterionellopsis glacialis*	4.4	3.2
*Bacillariaceae*	−1.8	0.4
*Biddulphia* spp.	1.3	2.4
*Cerataulina* spp.	0.2	−1.3
*Chaetoceros* spp.	0.01	0.01
*Ciliophora*	3.5	4.1
*Cryptophyceae*	2.7	6.2
*Dactyliosolen fragilissimus*	−1.2	−5.2
*Ditylum* spp.	7.6	6.5
*Euglenaceae*	3.0	−2.1
*Guinardia delicatula*	−7.7	−5.1
*Guinardia striata*	2.5	−0.4
*Gymnodiniaceae+Gymnodinium* spp.	4.3	4.3
*Leptocylindrus* spp.	−4.5	−1.6
*Melosiraceae*	−5.3	−7.2
*Navicula+Fallacia+Haslea+Lyrella+Petroneis* spp.	1.4	1.3
*Nitzschia longissima*	−3.3	−2.2
*Odontella* spp.	−0.2	−2.5
*Paralia sulcata*	−4.9	−1.1
*Plagiogramma* spp.	−5.1	4.4
*Pleurosigma+Gyrosigma* spp.	4.1	5.7
*Phaeocystis* spp.	−11.3	−8.7
*Prorocentrum* spp.	4.2	3.2
*Pseudo-nitzschia* spp.	1.7	4.7
*Rhizosolenia imbricata+styliformis*	4.9	3.2
*Rhizosolenia setigera+pungens*	−5.5	1.7
*Scrippsiella+Ensiculifera+Pentapharsodinium+Bysmatrum* spp.	−3.5	−6.9
*Skeletonema costatum*	0.01	0.01
*Thalassionema nitzschioides*	−1.5	1.1
*Thalassiosiracaea*	2.0	−3.4

Groups were determined as described in the Material and Methods. Coefficients represent the contributions of each group to the total functional response of the DEB model, given by variation in length or DFM.

Relative contribution coefficients associated with DFM/length variations ranged from 0.076/0.065 for *Ditylum* spp. to −0.113/−0.087 for *Phaeocystis* spp; 57% of them were positive. Contributions obtained with shell lengths or DFM were not significantly different (Kolmogorov-Smirnov test, p>0.05). Consensus on the sign of the contributions was 73%. Species ranking was similar for length and DFM.

*Cerataulina* spp., *Euglenaceae, Guinardia striata* and *Thalassiosiraceae* play a positive role in growth in terms of mass but not in terms of length. On the contrary, *Bacillariaceae*, *Plagiogramma* spp, *Rhizosolenia setigera*, *R. pungens* and *Thalassionema nitzschioides* seem to have a positive effect on growth in terms of length, but not in terms of mass.

**Figure 7:**
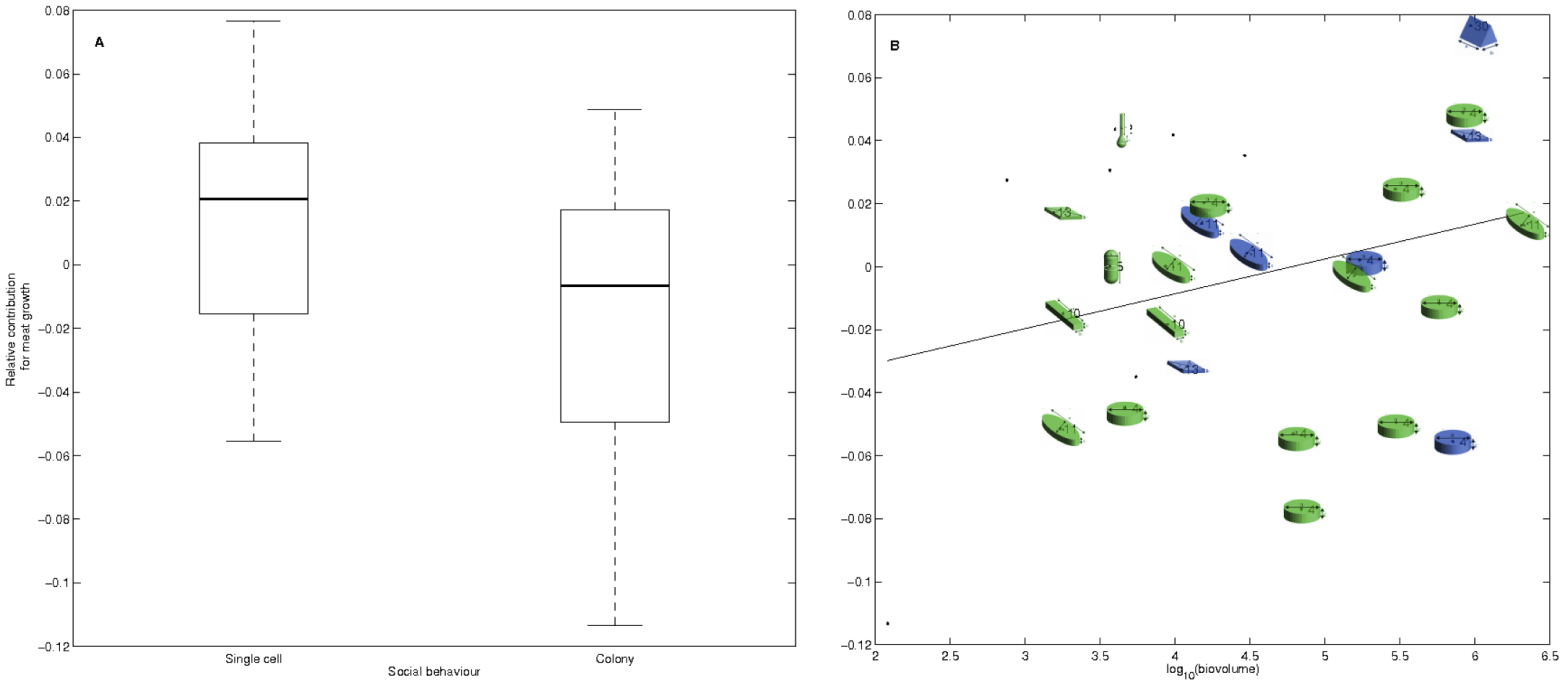
Effect of social behaviour (A) and biovolume (B) on mussel preference for plankton species. In the second panel, shapes correspond to the shape classification of each species, according to [47]. Blue color indicates that a species is free-living; green color indicates that it tends to form colonies. Black dots correspond to species with no defined shape.

Both distributions were normal (Shapiro-Wilk test, p>0.05), enabling the use of the ANCOVA method. Social behavior has a significant effect on food quality, related to DFM (ANCOVA, F_1,27_ = 2.9, p<0.01), while this effect is unclear for length-related coefficients (ANCOVA, F_1,27_ = 2.8, p = 0.11). When plankton species tend to form colonies, mussel affinity decreases (Fig. 7A). Biovolume was not deemed significant in either group of contributions (ANCOVA, F_1,27_ = 2.2, p = 0.15 for DFM and F_1,27_ = 0.4, p = 0.53 for length). However, a slight effect can be seen, as mussels seem to grow better on larger cells (Fig. 7B). Shape effect was not significant in the analysis; however, it can be seen that large species with low contributions are all cylindrical. Among the positive contributions, 23% of plankton groups are cylinder-shaped, but these account for 46% of negative contributions. When this cylindrical shape is excluded from the analysis, size effect is significant at the 0.1 level.

## Discussion

### DEB performance

Mussel flesh and shell length variations reflect the ecosystem dynamics and seem to magnify them. The first mass increase (+135% in March and +85% at the end of summer) was higher than observed elsewhere for intertidal mussels, even on larger individuals [9], [52], [53]. Maximum mass at the end of the summer was surprisingly high for mussels of this size and age [4], [31], [54]. Relative gain remained coherent as it was still lower than values obtained with continuously immersed mussels [7], [55]. In all these cases, mass gain was observed much later in the year, with the more favorable climatic conditions. Total mass loss (58%) recorded in 2011 was among the highest values obtained during starvation studies [5], [6], [31].

The DEB model, as it is presently parameterized, is not able to reproduce these observations. Different parameter sets and food proxies have been tried but it has not been possible to obtain less than a 20% deviation in mass simulation, except when the input was made up of *Chaetocero*s spp only. However, diet diversity clearly improves the fit of the simulation to the observations when chl a was balanced by species richness (Table 2). These contrasted results are the first step towards a more specific analysis of the different plankton species.

More importantly, bias was always the same at the beginning of the simulation, underestimating mass during 2010. In 2011, only a total absence of food could lead to mass loss. Furthermore, while simulations were more satisfactory with the set of parameters from [13] for the first year, the one from [6] was more efficient at modeling the mass loss in 2011. When trying to avoid this problem by reconstructing the functional response, we found values over 7 for the beginning of 2010. Cardoso *et al.* [27] were faced with the same problems with *Macoma balthica* modeling, leading to functional responses higher than 1. We can conclude, as they did, that work is still needed on parameterization. δ and *X_k_* are already known to depend on study site [5], [6] but other parameters may also be sensitive to phytoplankton ecotypes. We should therefore focus more on variations than on values.

Model quality is also problematic in recent works on *M. edulis*: while shell length is often correctly reproduced, this is not the case for DFM [7], [8] although mussel flesh is the most important aspect from an economic production viewpoint. The higher variability of flesh mass is mostly due to the losses that cannot be reproduced in shell length variation as this is an exoskeleton that is made of metabolically inert material. Gamete and reserve loss can be modeled with DEB theory, but structural loss cannot be modeled without altering the relationship between length and somatic energy [6]. This is due to the fact that we only have access to shell length and not body length. Studies have found no correlation between flesh and shell growth, even when both are increasing [12], [56]. This is partly due to differences in timing: shell length growth may precede soft tissue growth ([57], our data set) or succeed it [56]. Shell material is different from soft tissues and part of it comes from non-metabolic sources [58], [59]. This explains observed shell growth during starvation periods [60]. For the moment, the DEB model assumes that shell length is directly linked to structural flesh growth but, if this correlation does not always hold, other parameters will also need to be re-evaluated to obtain further knowledge about the species (*e.g.*, investment ratio κ). This also calls into question the use of shell length alone in functional response computation, as it can lead to the overestimation of ambient food conditions [26]. On the contrary, a comparison of both length and mass should be performed before conclusions are drawn. This is all the more difficult as shell length monitoring is preferable to avoid sampling bias that could emerge from the killing of animals to measure body length and dry flesh mass.

### Food preferences

The first DEB modeling led us to choose several food quantifiers to test. We focused on phytoplankton abundance which was the closest available approximate of primary production. Chl a is commonly used, but its production inside each cell depends on varying environmental conditions [61]. POC and POM are composite elements that may overestimate available food [53]. Finally, Bracken *et al.* have found that mussels might depend more on phytoplankton than on other organic elements [62]. Plankton abundance was also the most flexible variable, allowing for several levels of detail. Pre-processing included the use of a transformation to homogenize values, which can be highly variable. We chose a Holling-type II transformation (eq. 3) mostly for its physiological grounding and closeness to the DEB formulation.

It is difficult to compare our plankton dataset with others in the literature because plankton assemblages and successions are highly variable. However, some species seem to bring about a consensus. For instance, our results agree with [24], showing that *Leptocylindrus* is not ingested by mussels, while *Pleurosigma* and *Gyrosigma* spp. are preferentially ingested and *Nitzschia longissima* and *Thalassiosiraceae* may have neutral roles.

Underlying patterns appeared among food preferences. Free-living cells seem to have a positive effect on mussel growth. Even if not significant in our dataset, a preference for larger species may be another component of food quality. Both characteristics may tend towards a passive selectivity, relying both on physical and chemical properties. Size is important, as put forward by [63], who used the relative amount of small planktonic species as a depletion indicator because it is the only part of plankton that cannot be affected by bivalve consumption. Following [63] and [20], picoplankton was ignored and addition of nanoplankton to our model did not improve the fit. Even when targeting species in an accurate size range according to [20], smaller species were still less important for mussel growth. Cell size is modified by the ability to form a colony, increasing the actual volume that is filtered by mussels in the field. Our results could therefore be seen as contradictory.

The mechanism behind size preference needs to be clarified, as variability in retention efficiency cannot be attributed to larger species size only [20]. Colonies, which rely on other chemical components to bind themselves together, may overload the digestive system or the ciliary-gill pump, or may clog the gills [53]; this would trigger the ejection of pseudofaeces and/or feces consisting of undigested material [17]. Such a mechanism could explain the food assimilation decrease and mass loss, and seems all the more probable as *Phaeocystis* spp., forming colonies surrounded by an organic mucilage that can decrease clearance rate [64], are identified as the worst food source in our dataset. The potential role of shape also needs to be investigated.

Other plankton life history traits were not considered to make significant contributions to food quality, which may indicate the importance of a diverse diet. Regarding plankton ecological niche, Rouillon *et al.* [21] found more tychopelagic species in mussel stomachs than in ambient water, and Lefebvre *et al.* [65] showed that oyster growth in the Bay of Veys was dependent on microphytobenthos. Toupoint *et al.* [66] pointed out that pelagic cues overwhelmed biofilm ones, at least for mussel settlement. Our study cannot settle this argument, as few benthic species were found in our dataset and no biofilms could be observed at our sampling site. These films may not settle because of water mixing and sampling during flood tide leads to a bias towards pelagic species.

No preference was found for diatoms or dinoflagellates. The proportion of diatoms in food sources match that observed in the whole dataset. Previous studies are contradictory: some insist on the importance of diatoms in mussel diet [22], [67] while others emphasize the increase of dinoflagellates in the mussel diet compared with available species [68]; this has been discussed at length by Rouillon *et al.* [21]. Currently, our dataset can only show that both these groups are consumed and can play both positive and negative roles in food quality. Mussels may be sensitive to finer characteristics and/or may favor diversity [69].

Other characteristics that could have been investigated in our dataset include: how stickiness, electrostatic charge, mucopolysaccharides affect capture efficiency [17], and how morphology impacts palatability and digestibility [15]. Protein, carbohydrate and lipid contents are the key to assessing food effect on mussel growth by changing its composition [18] and even metabolism and reproductive cycle [70], [19]. Plankton composition is, however, too variable, and time- and site-dependent [14], [43], [62] to use values from the literature. Work on these aspects remains to be done and would certainly help in the search to find a structure behind our local species results. Once again, variability is high and the species that can be found in our area are certainly missing from the nearby sea [71] or will be in the coming years [30].

Finally, there are other food sources that have not been investigated and have already been found in the mussel diet: zooplankton [72], crustaceans, cnidarians, nematodes [22] and detritus [25] also contribute to the organic matter that can be ingested. Recent results tend to show that all of these sources are less influential than diatoms [23], but spatial and temporal variability would likely moderate any general conclusion [12].

Until now, we have tried to explain mass gain and loss with an emphasis on food availability and quality. Metabolism may have been altered by a significant switch between abundance and restriction. The former may have led to a decrease in growth efficiency that worsened the effect of restriction [73]. A high concentration of PIM may have altered assimilation efficiency while food was already low in quantity and quality for mussels. However, two other, non-exclusive, explanations should be considered but cannot be proven with our dataset.

The reproductive cycle must have played a key role in mass regulation [54]. We cannot differentiate the loss due to starvation from that due to spawning. These must have coexisted as spawning alone cannot explain the mass loss in its entirety. Gonadosomatic ratio, although very variable, rarely reaches 55% [74], [75], [76], and recent studies tend to show that *M. edulis* might invest less in reproduction than was previously thought [13]. No mass recovery was recorded for 5 months, contrary to what is usually observed after spawning [56]. Conversely, this mass drop is too sharp to be due to metabolism alone.

Mussels have multiple and contradictory reproductive strategies depending on environmental conditions [19], [77], [78]. According to mussel farmers working in the study area, climatic conditions were very favorable to spawning in 2011. Metabolism might change during spawning time and requires more energy [79] or energy in a different form [24], which could have worsened mass loss that year. Loss of mass due to spawning in the DEB model depends on several parameters: allocation parameter κ, spawning efficiency and percentage of gametes left in the reproductive compartment after spawning. The latter has been discussed: mussels can spawn completely [37] or partially [76]. Furthermore, the use of GSI and temperature thresholds was not successful in reproducing growth and led to incoherent patterns (*e.g.* up to 5 or 6 spawning dates in 2010 at times where the actual reproduction cycle was not completed). This was not the main point of our study, which is why we decided, like [7] and [12], to empty the gonads at a fixed spawning date. Without further organ differentiation, it would have been presumptuous to model a reproductive cycle for our experiment, which is why this explanation has not been developed further; knowledge is still required in this area and needs to be improved.

The possibility of infection must also be presented. During 2010, the presence of *Mytilicola intestinalis* was recorded on Utah Beach. Although infestation level was ranked low, there is still a possibility that the parasite developed and infected mussels in our experiments. Digestive disruptions may have resulted, decreasing assimilation efficiency; maintenance costs may also have been increased by the presence of a parasite. This could lead to differences in the physiological parameters describing mussel growth and explain the problems in DEB simulations that were observed even when food input was reconstructed.

To conclude, this article highlights the difficulty of representing different mussel growth patterns with a model smoothing tendency. Work needs to be done on DEB parameterization for *M. edulis* in this area. In the absence of further information, functional response reconstruction enabled us to get over the problem while still taking into account other environmental elements. This led to the selection of the preferred species that mussels have in the environments and to the identification of some patterns. A size gradient is noticeable in our dataset. Mussels tend to grow better on larger, single species. Plankton composition now needs to be studied further in order to relate it to mussel growth and investment.

## Supporting Information

Figure S1 **Comparisons of observed and modeled DFM as a function of cubed length.**
(TIFF)

Figure S2 **Example of an eigenvector contribution to the model coefficients for the smallest eigenvalue, using** a model optimizing the scaling coefficient c_i_ and simultaneous contributions of a_i_ and b_i_.
(TIFF)

Figure S3 **Evolution of the parameter identifiability with the number of species taken into account.** The condition number of the Hessian matrix of the model cost function (in grey) is related to the convergence rate of the model. The fit is measured by the mean square model error (in black).
(TIFF)

Table S1 **Plankton ecological characteristics.** Family affiliation and social behavior are from [44]. Position in the water column is mostly from [45] and [28]. Habitats are from [48], [49], [50] and [51]. Biovolumes are from [46].
(DOC)

Text S1 **Main DEB model equations.**
(DOC)

Text S2 **Analysis of the food quality assessment model.**
(DOC)
