# Supplementary figures and images for: Towards the Determination of Mytilus edulis Food Preferences Using the Dynamic Energy Budget (DEB) Theory
Source: PLoS One. 2014 Oct 23;9(10):e109796. doi: 10.1371/journal.pone.0109796 (PMC4207687; doi:10.1371/journal.pone.0109796)

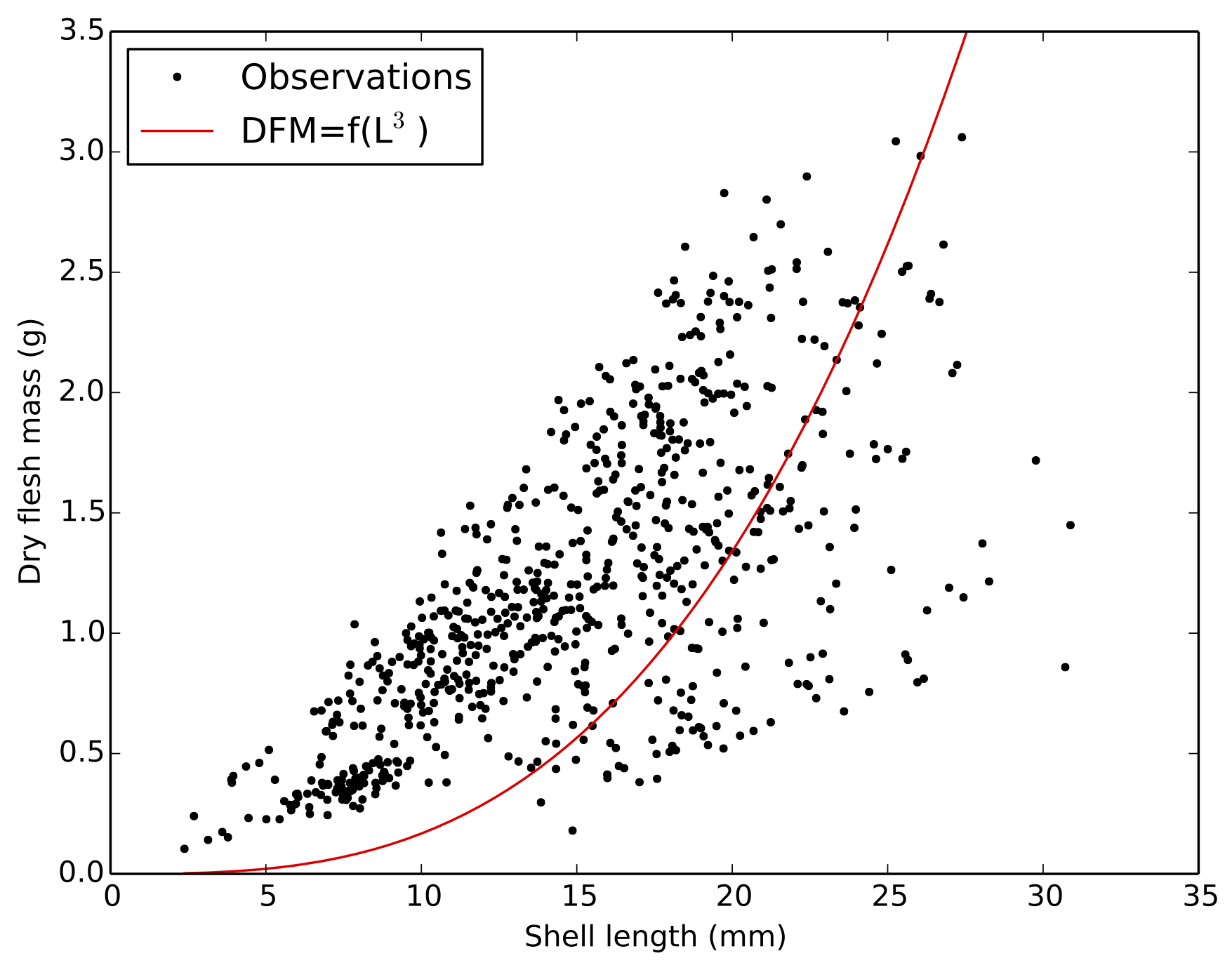

Supplement: Figure S1 — Comparisons of observed and modeled DFM as a function of cubed length. (TIFF) [file pone.0109796.s001.tiff]

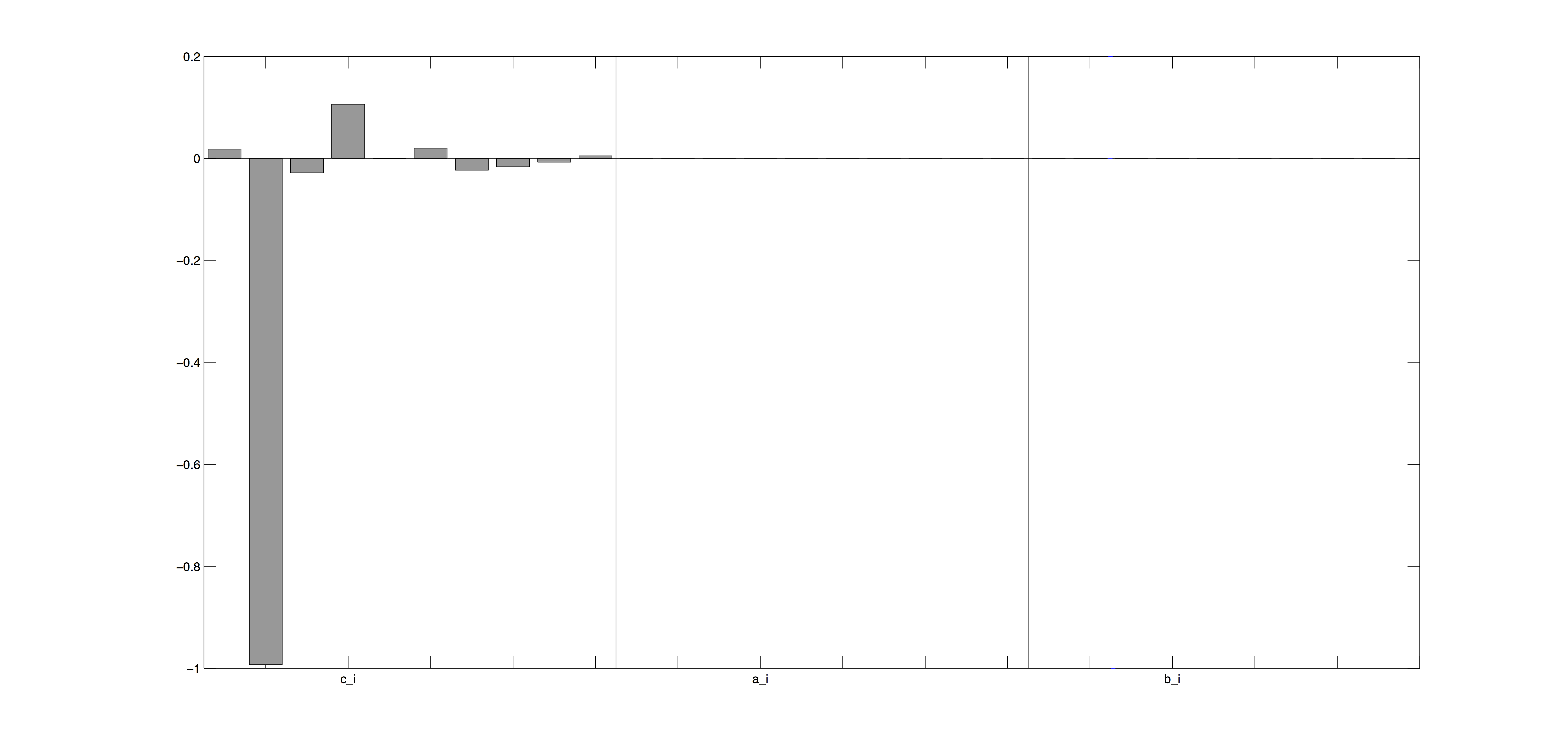

Supplement: Figure S2 — Example of an eigenvector contribution to the model coefficients for the smallest eigenvalue, using a model optimizing the scaling coefficient ci and simultaneous contributions of ai and bi. (TIFF) [file pone.0109796.s002.tiff]

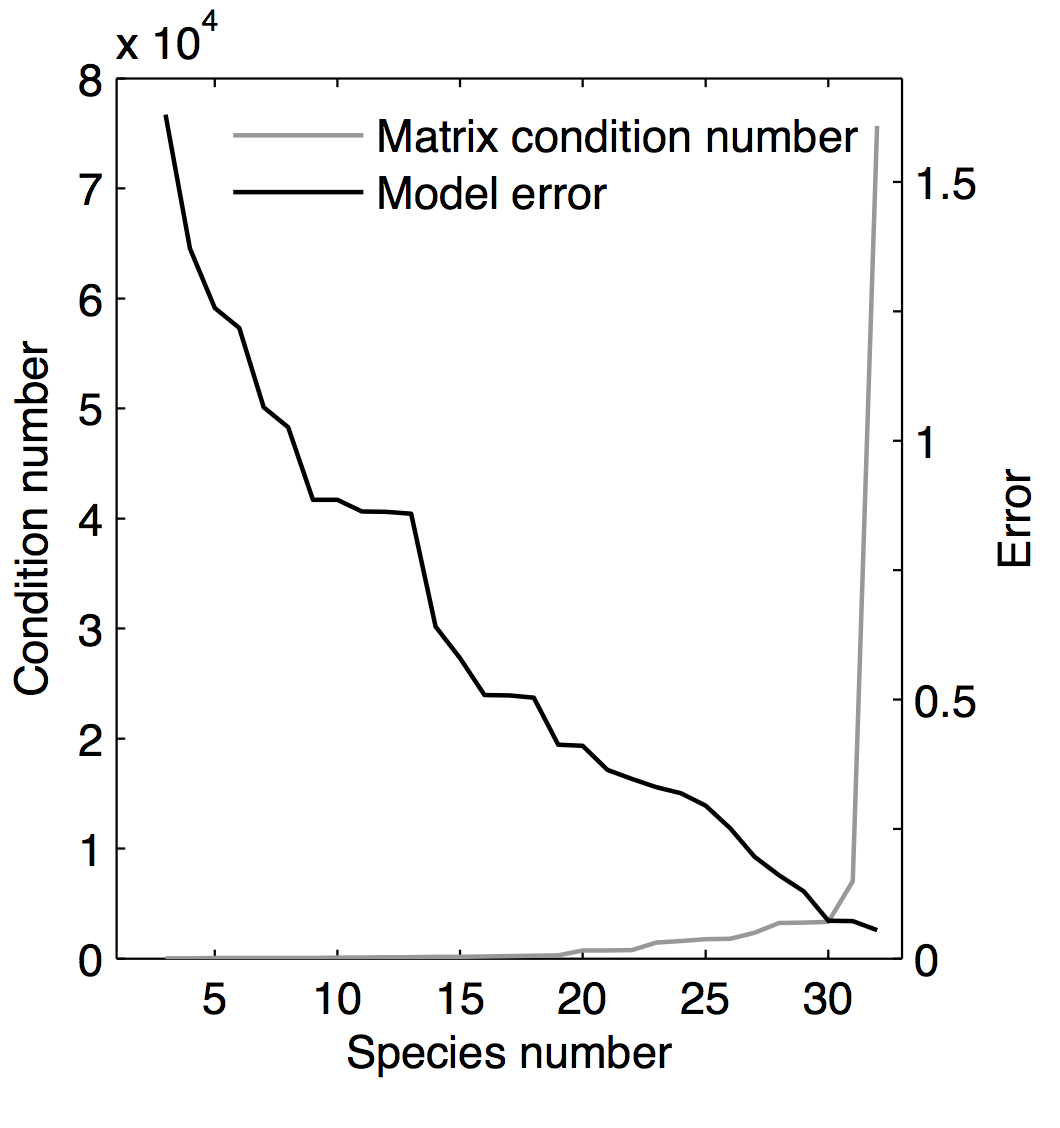

Supplement: Figure S3 — Evolution of the parameter identifiability with the number of species taken into account. The condition number of the Hessian matrix of the model cost function (in grey) is related to the convergence rate of the model. The fit is measured by the mean square model error (in black). (TIFF) [file pone.0109796.s003.tiff]
