# Supplementary material for: Towards the Determination of Mytilus edulis Food Preferences Using the Dynamic Energy Budget (DEB) Theory
Source: PLoS One. 2014 Oct 23;9(10):e109796. doi: 10.1371/journal.pone.0109796 (PMC4207687; doi:10.1371/journal.pone.0109796)
Supplement: Table S1 — Plankton ecological characteristics. Family affiliation and social behavior are from [44]. Position in the water column is mostly from [45] and [28]. Habitats are from [48], [49], [50] and [51]. Biovolumes are from [46]. (DOC) [file pone.0109796.s004.doc]

Table S1 Plankton ecological characteristics

| **Plankton group** | **Group** | **Social behavior** | **Habitat** | **Position in the water column** | **Biovolume**  **(μm^3^)** | **Shape** |
| --- | --- | --- | --- | --- | --- | --- |
| *Asterionellopsis glacialis* | diat (P) | colony | coastal/oceanic | tychopelagic | 4006 | cone + half spere + cylinder |
| *Bacillaria*ceae | diat (P) | colony | coastal/oceanic | tychopelagic | 8875 | rectangular box |
| *Biddulphia* spp. | diat (C) | colony | oceanic | benthic | 2122539 | prism on elliptic base |
| *Cerataulina* spp. | diat (C) | single cell | coastal | pelagic | 170127 | cylinder |
| *Chaetoceros* spp*.* | diat (C) | colony | coastal/oceanic | pelagic | 8614 | prism on elliptic base |
| *Ciliophora* | other | single cell | coastal/oceanic | tychopelagic | 29218 |  |
| *Cryptophyceae* | other | single cell | coastal | pelagic | 755 |  |
| *Dactyliosolen fragilissimus* | diat (C) | colony | coastal/oceanic | pelagic | 578273 | cylinder |
| *Ditylum* spp. | diat (C) | single cell | coastal/oceanic | pelagic | 938772 | prism on triangle base |
| *Euglenaceae* | other | single cell | coastal | undetermined | 3666 |  |
| *Guinardia delicatula* | diat (C) | colony | oceanic | pelagic | 69618 | cylinder |
| *Guinardia striata* | diat  (C) | colony | oceanic | pelagic | 295702 | cylinder |
| *Gymnodiniaceae+ Gymnodinium* | dino | single cell | coastal/oceanic | tychopelagic | 30130 | prism on elliptic base |
| *Leptocylindrus* spp. | diat (C) | colony | coastal | pelagic | 4614 | cylinder |
| *Melosiraceae* | diat (C) | colony | coastal | tychopelagic | 61163 | cylinder |
| *Navicula + Fallacia + Haslea + Lyrella + Petroneis* spp. | diat (P) | single cell | coastal | tychopelagic | 15029 | prism on elliptic base |
| *Nitzschia longissima* | diat (P) | single cell | coastal/oceanic | benthic | 10925 | prism on parallelogram base |
| *Odontella* spp. | diat (C) | colony | coastal/oceanic | pelagic | 141666 | prism on elliptic base |
| *Paralia sulcata* | diat (C) | colony | coastal/oceanic | tychopelagic | 298723 | cylinder |
| *Phaeocystis* spp. | dino | colony | coastal/oceanic | tychopelagic | 121 |  |
| *Plagiogramma* spp. | diat (C) | colony | coastal/oceanic | tychopelagic | 1711 | prism on elliptic base |
| *Pleurosigma + Gyrosigma* | diat (P) | single cell | coastal/oceanic | benthic | 848160 | prism on parallelogram base |
| *Prorocentrum* spp. | dino | single cell | coastal/oceanic | tychopelagic | 9702 |  |
| *Pseudo-nitzschia* spp. | diat (P) | colony | coastal/oceanic | pelagic | 1556 | prism on parallelogram base |
| *Rhizosolenia imbricata + styliformis* | diat (C) | colony | oceanic | pelagic | 829690 | cylinder |
| *Rhizosolenia setigera + pungens* | diat (C) | single cell | oceanic | pelagic | 712209 | cylinder |
| *Scrippsiella + Ensiculifera + Pentapharsodinium + Bysmatrum* spp. | dino | single cell | coastal/oceanic | tychopelagic | 5480 |  |
| *Skeletonema costatum* | diat (C) | colony | coastal | pelagic | 3669 | cylinder + 2 half-spheres |
| *Thalassionema nitzschioides* | diat (P) | colony | coastal | pelagic | 1790 | rectangular box |
| *Thalassiosiracaea* | diat (C) | colony | coastal/oceanic | pelagic | 15650 | cylinder |

Plankton characteristics defined as described in Material and methods. Diat: diatoms; dino: dinoflagellates; P: pennate diatoms; C: centric diatoms.
