# Supplementary material for: Towards the Determination of Mytilus edulis Food Preferences Using the Dynamic Energy Budget (DEB) Theory
Source: PLoS One. 2014 Oct 23;9(10):e109796. doi: 10.1371/journal.pone.0109796 (PMC4207687; doi:10.1371/journal.pone.0109796)
Supplement: Text S1 — Main DEB model equations. (DOC) [file pone.0109796.s005.doc]

**Text S1. Main DEB model equations**

This appendix summarizes the main equations used in the DEB model according to Rosland *et al.* [6] and Saraiva *et al.* [13]. DEB symbols and notations are the same as in [1] where brackets [ ] denote quantities per unit structural volume and braces { } denote quantities per unit surface area of the structural volume.

In Saraiva’s implementation, feeding mechanisms are described in four steps: water is cleared (1), its content is then filtered (2), ingested (3) and assimilated (4).

$$\begin{matrix} \mathop{C}\limits^{.} _{R}=\frac{\left\{\mathop{C}\limits^{.} _{Rm}\right\}}{1+\sum_{i} \frac{\lambda_{i}F_{i}\left\{\mathop{C}\limits^{.} _{Rm}\right\}}{\left\{\mathop{\text{J}}\limits^{.} _{FiFm}\right\}}}V^{2/3}(1) \\ \mathop{\text{J}}\limits^{.} _{FiF}=\mathop{C}\limits^{.} _{R}\lambda_{i}F_{i}(2) \\ \mathop{\text{J}}\limits^{.} _{FiI}=\frac{\rho_{i}\mathop{\text{J}}\limits^{.} _{FiF}}{1 +\sum_{j} \frac{\rho_{j}\mathop{\text{J}}\limits^{.} _{FjF}}{\mathop{\text{J}}\limits^{.} _{Fi\mathrm{Im}}}}(3) \\ \mathop{p}\limits^{.} _{A}=C\cdot AE\cdot\mathop{\text{J}}\limits^{.} _{FI}(4) \end{matrix}$$

where C_R_ is the amount of cleared water, {C_Rm_} is the maximum clearance rate, λ_i_ is a conversion factor from food units to mol C, Fi is the food proxy (in our model, two variables were used: a classical food proxy such as chl a or phytoplankton abundance and a fixed fraction of TPM to account for inorganic material). $\left\{\mathop{\text{J}}\limits^{.} _{FiFm}\right\}$ and $\mathop{\text{J}}\limits^{.} _{Fi\mathrm{Im}}$ are the maximum filtration and ingestion rate for food type *i,* respectively while*,* $\mathop{\text{J}}\limits^{.} _{FiF}$and $\mathop{\text{J}}\limits^{.} _{FiI}$are the actual filtered and ingested food. ρ_i_ is the food binding probability. $\mathop{p}\limits^{.} _{A}$ is the assimilation rate, depending on a conversion factor (from mol to J), and AE is the ingestion rate.

In Rosland’s implementation, used for functional response assessment, assimilation $\mathop{p}\limits^{.} _{A}$ is proportional to the surface of the structural body *V^2/3^* and to the maximum ingestion rate *{p_Xm_}*, corrected for thermal effects.

$\mathop{p}\limits^{.} _{A}=AE\left\{\mathop{p}\limits^{.} _{Xm}\right\}fV^{2/3}$ (5)

with

$f=\frac{F}{F+ X_{k}}$ (6)

where *f* is the functional response to ambient food *F* standardized with the half-saturation coefficient *X_k_*.

In all cases, energy input leads to a change in the reserve compartment.

$\frac{dE}{dt}=\mathop{p}\limits^{.} _{A}-\mathop{p}\limits^{.} _{C}$(7)

where $\mathop{p}\limits^{.} _{C}$ is the catabolic flux due to growth and maintenance costs.

(8)

where [E] is the energy density ([E]=E/V), [E_G_] represents the volume-specific costs of structural growth, κ is the allocation coefficient and [E_m_] is the maximum storage density.

Energy is then redistributed to structural growth and reproduction.

$\frac{dV}{dt}=\frac{(\kappa\mathop{p}\limits^{.} _{C}-\mathop{p}\limits^{.} _{M})}{\left[E_{G}\right]}$(9)

$\frac{dE_{R}}{dt}=(1-\kappa)\mathop{p}\limits^{.} _{C}-\frac{1-\kappa}{\kappa}\mathop{p}\limits^{.} _{M}$ (10)

When energy input is not sufficient for structural costs, lysis is possible.

$\frac{dE_{R}}{dt}=\kappa\mathop{p}\limits^{.} _{C}-\mathop{p}\limits^{.} _{M}$(11)

When the reproductive buffer is empty, structural meat can be decreased.

$\frac{dV}{dt}=\frac{(\kappa\mathop{p}\limits^{.} _{C}-\mathop{p}\limits^{.} _{M})}{C}$(12)

Finally, energy buffers and length can be related with a shape coefficient δ.

(13)
