# Supplementary material for: Towards the Determination of Mytilus edulis Food Preferences Using the Dynamic Energy Budget (DEB) Theory
Source: PLoS One. 2014 Oct 23;9(10):e109796. doi: 10.1371/journal.pone.0109796 (PMC4207687; doi:10.1371/journal.pone.0109796)
Supplement: Text S2 — Analysis of the food quality assessment model. (DOC) [file pone.0109796.s006.doc]

**Text S2. Analysis of the food quality assessment model**

This appendix analyses the model used to calculate the contribution of each plankton species to mussel length and dry flesh mass growth. This is represented by the set of equations (1):

$\left\{\begin{matrix} \sum_{i} a_{i}\frac{F_{i,t}}{F_{i,t}+c_{i}}=f_{l,t} \\ \sum_{i} b_{i}\frac{F_{i,t}}{F_{i,t}+c_{i}}=f_{m,t} \end{matrix}\right.$(1)

$d=\frac{1}{2}\sum_{t} \left[(\sum_{i} a_{i}\frac{F_{i,t}}{F_{i,t}+c_{i}}-f_{l,t})^{2}+(\sum_{i} b_{i}\frac{F_{i,t}}{F_{i,t}+c_{i}}-f_{m,t})^{2}\right]$ where *a_i_* and *b_i_* represent the contribution of the food source *F_i_* to the function response *f_l/m_* reconstructed with length or mass, respectively, and *c_i_* is a scaling coefficient. This set of equations applies to one sampling date. The cost function that needs to be minimized is given by eq. 2.

(2)

The model quality was assessed by running both identifiability and sensitivity analyses. The hessian matrix [39], composed of the second derivatives of the cost function with respect to its parameters, was calculated near the global minimum. The matrix condition number is an indicator of the convergence rate of the model. Large values indicate that the model is poorly formulated and cannot be used to determine all parameters at the same time. Eigenvectors and eigenvalues provide more detailed information on the most uncertain parameters.

A first evaluation of the whole model showed that the condition number was above 10^13^ for less than 10 species, indicating poor parameter identifiability. Eigenvectors corresponding to the lowest eigenvalues all had significant contributions for scaling coefficients. Fig. S2 shows the values of the elements that compose the eigenvector corresponding to the lowest eigenvalue. This vector contributed only to the scaling coefficients. These coefficients were thus excluded from the optimization and replaced by the median value of the abundance of each species when it was measured on the field. This value may seem arbitrary but is close to what can be computed when using this species as a food input directly in the DEB model. This can be seen as a first step towards a better formulation with a larger dataset.

A sensitivity analysis was then launched for parameters that were excluded from the optimization process. Scaling coefficients were varied around their fixed value by ±20%. This led to less than 10% variation in the final plankton contributions, no change in the sign of any contribution or in the ranking of species with extreme contributions (best or worst five species for mussel growth). For least influent species, change in ranking was never more than two ranks. This tends to confirm that this choice in scaling coefficients did not alter the results.

When using this formulation, the condition number of the matrix was greatly improved. Fig. S3 shows the increase of the condition number with increasing richness (in grey), while the mean squared error decreases (in black). The final number of species is a result of a trade-off between a low condition number (below 10^4^, as in [40], [41] and [42]) and error, and a high number of species in order to detect structuring ecological components. With 30 species, the condition number is 3322 and error is around 0.07.
